# Understanding the Role of Mass-Unloading in a Filament Eruption

**DOI:** 10.1007/s11207-017-1224-y

**Published:** 2018-01-03

**Authors:** J. M. Jenkins, D. M. Long, L. van Driel-Gesztelyi, J. Carlyle

**Affiliations:** 1 Mullard Space Science Laboratory, University College London, Holmbury St. Mary, Dorking, Surrey RH5 6NT UK; 2 LESIA-Observatoire de Paris, CNRS, UPMC Univ Paris 06, Univ. Paris-Diderot, 92195 Meudon Cedex, France; 3 Konkoly Observatory of the Hungarian Academy of Sciences, Budapest, Hungary; 4 European Space Agency, ESTEC, Noordwijk, Netherlands

**Keywords:** Coronal mass ejections, initiation and propagation, Prominence, quiescent, Magnetic-fields, photosphere

## Abstract

We describe a partial filament eruption on 11 December 2011 that demonstrates that the inclusion of mass is an important next step for understanding solar eruptions. Observations from the *Solar Terrestrial Relations Observatory-Behind* (STEREO-B) and the *Solar Dynamics Observatory* (SDO) spacecraft were used to remove line-of-sight projection effects in filament motion and correlate the effect of plasma dynamics with the evolution of the filament height. Flux cancellation and nearby flux emergence are shown to have played a role in increasing the height of the filament *prior* to eruption. The two viewpoints allow the quantitative estimation of a large mass-unloading, the subsequent radial expansion, and the eruption of the filament to be investigated. A 1.8 to 4.1 lower-limit ratio between gravitational and magnetic-tension forces was found. We therefore conclude that following the loss-of-equilibrium of the flux-rope, the radial expansion of the flux-rope was restrained by the filamentary material until 70% of the mass had evacuated the structure through mass-unloading.

**Electronic Supplementary Material:**

The online version of this article (10.1007/s11207-017-1224-y) contains supplementary material, which is available to authorised users.

## Introduction

Multi-wavelength observations of the solar atmosphere reveal different features and phenomena depending on the emission/absorption characteristics of the material that is being observed. Frequently studied large-scale features include active regions, flares, filaments and prominences, and coronal mass ejections (CMEs). Eruptive activity on the Sun is largely associated with flares and CMEs, believed to be triggered by non-ideal and ideal instabilities, respectively. For a more detailed summary of solar features and their eruptions, see reviews by Forbes (2000), Webb and Howard (2012), Parenti (2014), and references therein.

Filaments and prominences show a minor preference to form with an average latitude of $\pm 25^{\circ} $ due to the presence of the highly sheared field across the polarity inversion line (PIL) of active regions, but they are also seen to exist at higher latitudes (Mackay, Gaizauskas, and Yeates, 2008; McIntosh *et al.*, 2014). Their difference in appearance is attributed to being different projections that are due to the parameters employed in observations, but they are fundamentally the same phenomena. Filaments are observationally identified as dynamic, dark, and elongated slab-like features against the disc and are thus seen in absorption. Observations of filaments require that the wavelengths used correspond to the wavelength that is either absorbed or volume-blocked (reflected) by the material present in the filament. Their off-limb counterparts, prominences, exhibit both similar and different plane-of-sky dynamics and features to filaments (Gunár and Mackay, 2015). Prominences are also observed as elongated structures, but are seen in emission protruding from the surface and appearing above the limb, requiring that the wavelengths used in observations correspond to the emission of the material contained within the prominence. Early spectroscopic observations of the solar atmosphere (*i.e.* above the photosphere) revealed the presence of highly ionised material such as Fe xiv (previously thought to be the new element coronium), requiring effective temperatures of $>10^{6}$ K (Edlén, 1943). Observations of off-limb prominences reveal that the observed material is in fact best seen in the H$\upalpha $ 6562.8 Å and He ii 304 Å passbands, requiring a much cooler effective plasma temperature of $10^{3}\,\text{--}\,10^{4}$ K, indicative of chromospheric material. The consensus is that filaments/prominences consist of this chromospheric plasma suspended in, and thermally isolated from, the hotter corona. For a more in-depth review of filament and prominence observations see Parenti (2014).

The suspension of this chromospheric plasma is potentially facilitated by a system of helical field lines. van Ballegooijen and Martens (1989) describe a model based on a dynamically formed system gradually becoming disconnected from the surface at points of local reconnection. This helical system is referred to as a flux rope and forms above a PIL. The evolution of this magnetically buoyant structure then depends on the relationship between the magnetic tension, the magnetic pressure gradient, and, if the structure contains plasma, gravity.

The magnetic models developed to study this loss-of-balance of forces internal to the flux rope are mainly based around the two ideal magnetohydrodynamic (MHD) instabilities: the kink and torus instabilities (*e.g.* Török and Kliem, 2005; Kliem and Török, 2006). The kink instability is thought to occur when the amount of internal twist in a flux rope, *i.e.* the number of turns in the field lines around the axis of the flux rope, exceeds some critical value. Such a highly wound flux rope then evolves to reduce this strong internal twist by transferring some of its twist into writhe (twist of the flux-rope axis), conserving helicity in the process (Hood and Priest, 1981). This increase in writhe includes an associated increase in the height and curvature of the apex of the flux rope, introducing a radial gradient in the magnetic pressure between the regions below and above the flux rope. If this gradient is sufficiently large (historically indicated by the value of the decay index $n_{\mathrm {c}}>3/2$, Bateman, 1978), the pressure gradient (hoop force) drives an exponential rise of the flux rope. The triggering of this exponential expansion due to the large gradient in the magnetic pressure force is referred to as the onset of the torus instability.

In addition to the internal evolution of the magnetic-field of a flux rope, the interaction with external magnetic structures and dynamics have been studied to probe their role in the evolution of a filament channel. Feynman and Martin (1995) and Chen and Shibata (2000) describe how the orientation of co-located flux emergence can have different effects on the stability of a nearby filament. Flux emergence manifests itself in the photospheric line-of-sight (LOS) magnetic-field data as opposite polarities that emerge, grow, and separate. The observational marker of emergence in the optical–extreme ultraviolet (EUV) wavelengths is often dynamic dark loops forming low in the chromosphere and expanding into the corona, whilst being heated and becoming bright. Although the initial emergence of flux typically contains the observational markers mentioned, the amount of flux that emerges and evolves can vary in scale. A more detailed description on the physics of flux emergence may be found in Cheung and Isobe (2014). The flux emergence associated with filament eruptions can either be located directly beneath the flux rope, as described by Palacios *et al.* (2015), or to the side. Emergence beneath the flux rope can trigger the eruption through tether-cutting, as described by Moore *et al.* (2001). Alternatively, emergence to the side of the flux rope can cause reconnection between the two systems to occur, such that the eruption is triggered by the weakening of the overlying field tension (Ding and Hu, 2008). Both of these scenarios are believed to be eruption triggers, whilst the eruption drivers are believed to still be instabilities such as the torus instability. The initial studies of the formation, stability, evolution, and eruption of filaments led the solar community to the conclusion that the evolution of the magnetic environment containing the filament was of primary importance. Démoulin (1998) described the need for intrinsic concave-up portions of the magnetic-field structure to be able to maintain the suspension of the plasma of a filament, as modelled by van Ballegooijen and Martens (1989), and simulated by authors such as Lionello *et al.* (2002) and Aulanier *et al.* (2010). This configuration can presumably be formed over a period of days or weeks and is thus in line with the time-line of filament formation according to observations. As a result, recent numerical models describing the destabilisation of flux ropes have focused mainly on the magnetic evolution, neglecting plasma processes associated with the filament contained within the flux rope. However, Démoulin (1998) also concluded that the plasma processes are capable of modifying the magnetic environment on timescales of an hour. Similarly, Gunár *et al.* (2013) examined non-linear force-free magnetic-dip models, concluding that the exclusion/inclusion of plasma could dramatically affect the *radial*-$B_{z}$ component of the dips within the magnetic field of a flux rope. This deformation of field lines through the addition or removal of plasma within the magnetic environment on short timescales is a key part of the mass-loading eruption mechanism described by Low (1996) and Klimchuk (2001). The mass-loading model suggests that a sufficiently large mass, such as the filamentary plasma, could allow the flux rope to build free magnetic energy without losing equilibrium until this mass is removed. Once the equilibrium is lost due to the unloading of the anchoring force supplied by the filamentary material, the magnetic environment would be free to expand (Forbes, 2000) and attempt to find a new equilibrium by increasing the height of the flux rope; however, this does not necessarily result in an eruption.

Unfortunately, the observations required to accurately correlate the effect of the magnetic environment and internal/external dynamics on the overall height–time (*h–t*) evolution of the flux rope are rare. However, the ideal conditions for drawing connections between filament mass dynamics and their relation to the *h–t* profile were demonstrated by Seaton *et al.* (2011). Their analysis of the event on 3 April 2010 describes an active-region filament observed from multiple perspectives. Observations from the *Atmospheric Imaging Assembly* (AIA: Lemen *et al.*, 2012) onboard the *Solar Dynamics Observatory* (SDO: Pesnell, Thompson, and Chamberlin, 2012) and the *Extreme Ultraviolet Imager* (EUVI: Wuelser *et al.*, 2004) onboard *Solar Terrestrial Relations Observatory-Behind* (STEREO: Kaiser *et al.*, 2008) isolated the *h–t* response to mass-flow dynamics and captured one of the first well-documented examples of the mass-loading eruption mechanism. Their event displayed mass motion towards the footpoints of the filament of interest *prior* to eruption. This unloading of filament mass was then seen to precede the increase in height of the filament spine until catastrophic loss-of-equilibrium set in and the eruption occurred. However, Seaton *et al.* (2011) did not quantify the degree of mass-unloading undergone in this event, nor did they quantitatively discuss this effect on the evolution of the entire filament.

Work completed by Bi *et al.* (2014) on the filament eruption that was of 23 February 2012 describes a filament eruption also seen from multiple perspectives. Using the opacity method and application outlined by Williams, Baker, and van Driel-Gesztelyi (2013) and Carlyle *et al.* (2014) respectively, the authors were able to estimate the amount of mass contained within the filament structure *prior* to eruption, and the degree of mass-unloading undergone with respect to the entire filament mass. Quantifying the degree of mass-unloading gives further insight into the physics at play but no conclusions on the energetics of the event are presented. Similarly the exact response of the *h–t* profile of the filament to the described plasma processes during the pre-eruption phase is omitted.

In this article, we discuss how the plasma within and magnetic field around a filament evolved *prior* to its partial eruption on 11 December 2011 at 05:53 UT. The event was observed by multiple observatories located at different points in the heliosphere, providing an opportunity to fully describe the evolution of the filament plasma. In Section 2 we present the outline of the article. In Section 3 we describe the observations made, before outlining the results of analysing these data in Section 4. The results are then interpreted and described in Section 5, before some conclusions are drawn in Section 6.

## Overview

**Figure 1:**
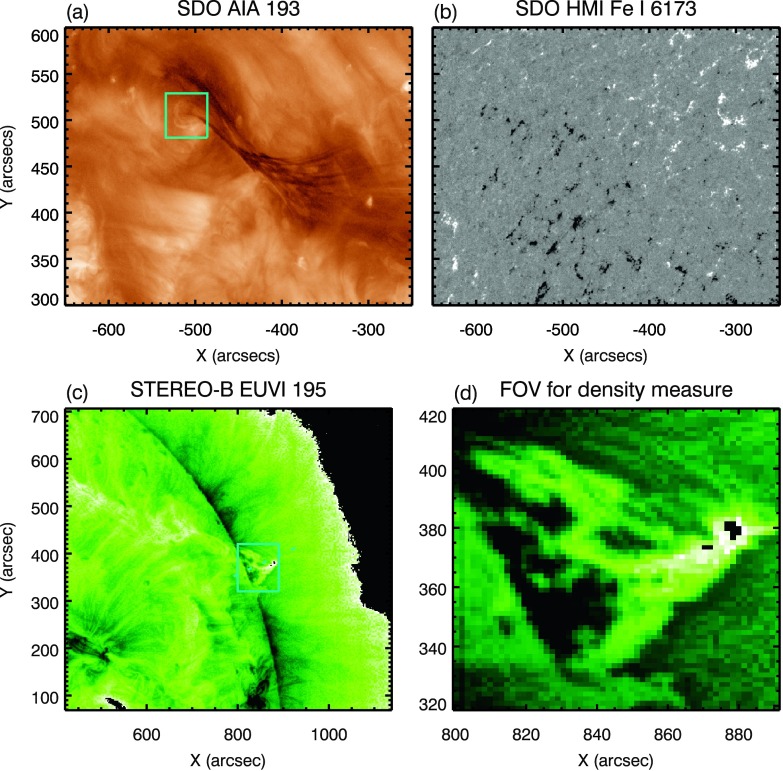
Filament and its photospheric magnetic-field environment as seen from the perspective of STEREO-B and SDO. Panel **a**: The filament as seen in the SDO/AIA 193 Å passband. The *cyan box* represents the FOV used for the density measures of Figure 7. Panel **b**: The same FOV as in **a** from the SDO/HMI instrument showing the LOS magnetic field saturated to $\pm 100$ G. Panel **c**: The filament (indicated by the *cyan box*) as seen on the limb by the STEREO-B/EUVI 195 Å passband using a reversed colour table. The *cyan-dashed line* indicates the location of the stack line for Figure 9. The *cyan box* represents the zoomed-in FOV shown in panel **d** and used for the density measures of Figure 8. All EUV images have the time stamp of 04:51 UT on 11 December 2011, and the STEREO-B/EUVI images have been processed using the multi-Gaussian normalisation technique (MGN: Morgan and Druckmüller, 2014).

In an effort to make the key events described in this article easier to follow, we now provide a brief summary of the observations made. The partial filament eruption studied here was observed on 11 December 2011 in the north-eastern quadrant of the solar disc by SDO and at the north-western limb by STEREO-B, as shown in Figure 1. The analysis described here focuses on the period leading up to the eruption, specifically from 12:00 UT on 10 December to 08:00 UT on 11 December.

**Figure 2:**
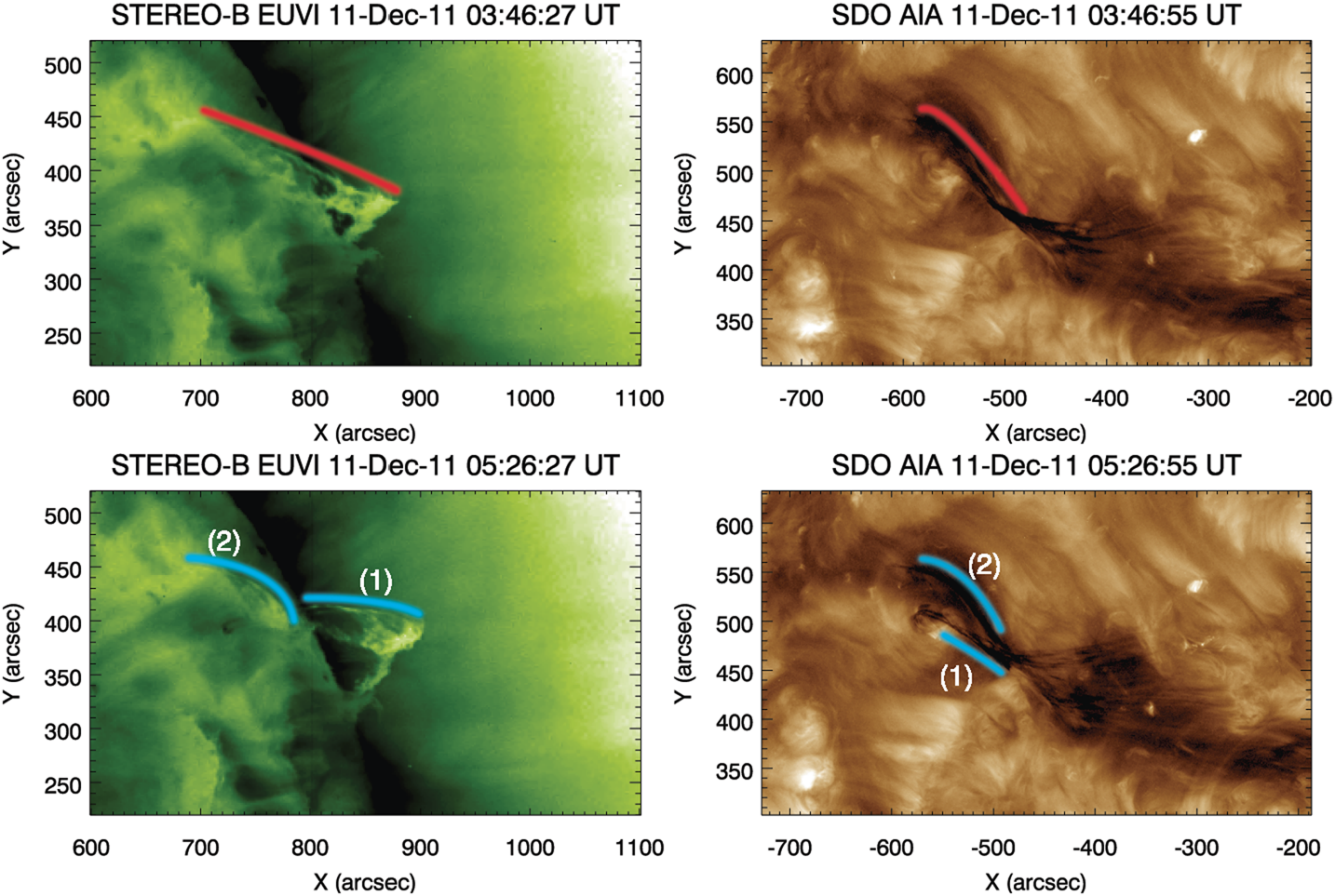
Splitting of the filament during its eruption as seen by STEREO-B (*left*, reverse colour table) and SDO (*right*). *Upper*: The *red line* traces the connection between the *upper* and *lower branches* of the filament (STEREO-B) and the south-western and north-eastern portions of the filament (SDO), indicating a magnetic connection highlighted by the illuminating material. *Lower*: The *blue lines* trace the edges of the two distinctly separated filament structures, (*1*) – dynamic portion (*2*) – restrained portion, just *prior* to the eruption of the dynamic portion of the filament. A movie of this figure accompanies the online version of this article in the Electronic Supplementary Material.

i) The filament of interest was one of several located in a large filament channel that spanned approximately half of the solar disc visible from SDO/AIA. LOS magnetic-field observations from the *Helioseismic Magnetic Imager* (HMI: Schou *et al.*, 2012) show that the filament channel was flanked by a very diffuse bipolar photospheric field, common for quiescent filament channels (Mackay, Gaizauskas, and Yeates, 2008). Approximately 18 hours before the eruption, flux cancellation was recorded along the PIL of this weak bipolar field. During this time, observations from STEREO-B/EUVI showed the filament of interest increasing in height. The flux cancellation along the PIL was then seen to have ceased approximately 12 hours before the eruption. This is discussed in more detail in Section 4.1.
ii) Approximately nine hours *prior* to the eruption, a small bipole was observed to emerge to the north-west of the filament. The orientation of the bipole was perpendicular to the axis of the filament channel. This small bipole grew in extent, reaching its peak value approximately five hours before the eruption of the filament, and subsequently it began to decay. As the bipole approached its peak strength, the filament ceased rising, remaining stationary for approximately one hour. This is discussed in Section 4.2.
iii) The filament was then seen to become unstable, potentially due to the associated flux rope becoming kink unstable, and it began a slow, exponential expansion through the corona. Observations from STEREO-B/EUVI suggest that the rising filament did not remain parallel to the surface; see Figure 2. Shortly after the expansion of the filament restarted, mass was observed flowing from the apex of the filament towards the north-eastern footpoint as observed by SDO. When the filament apex reached a height of approximately 65 – 70 Mm, approximately one and a half hours *prior* to the eruption, a large mass flow was observed draining from the apex down to the north-eastern footpoint of the filament. Shortly after the initiation of the large mass flow, the expansion of the filament dramatically accelerated. This is discussed in detail in Sections 4.3, 4.4, and 4.5.

During the eruption, the filament is seen to split in two, shown in Figure 2, suggesting that the magnetic structure containing the filament also split. As the higher, dynamic part of the filament reached a height of approximately 100 Mm, flare ribbons and two large EUV dimmings (Thompson *et al.*, 2000) that spanned supergranular boundaries formed in the low solar atmosphere, indicating a successful eruption of this portion of the filament. In addition to the brightenings on the surface, brightenings that appear to trace the outside of the magnetic structure suspending the filament are observed during the eruption, as shown in the online movie associated with Figure 2. The remaining portion of the split filament is visibly perturbed at this point but is unable to successfully erupt, ultimately reforming a part of the original filament a few hours later. Therefore the part of the filament that we have focused on in this study and used to define the eruption of the filament is the dynamic portion that successfully erupts into the heliosphere at 05:53 UT on 11 December 2011.

## Observations

SDO/AIA is an EUV imager that observes the Sun in ten passbands: seven EUV channels (94, 131, 171, 193, 211, 304, and 335 Å), two far-ultraviolet (FUV) channels (1600 and 1700 Å), and one visible channel (4500 Å) with a spatial resolution of $1.5^{\prime \prime }$. The 171 Å and 193 Å passbands in particular have peak emission temperatures of 0.6 and 1.3 – 2 MK, respectively, and thus primarily observe the upper chromosphere and low corona. HMI is another instrument onboard SDO designed to study the evolution of the photospheric magnetic-field and Doppler velocity. HMI images the Fe i absorption line at 6173 Å, with a spectral resolution of five points across the wings and core, and it characterises the effect of Zeeman splitting on emission originating at $\approx 100$ km above the photosphere (Fleck, Couvidat, and Straus, 2011). This produces an estimate of the LOS component of the photospheric field.

The STEREO mission consists of two identical non-Earth-orbit spacecraft. STEREO-A and STEREO-B are travelling ahead of and behind the Earth, respectively, and provide stereoscopic observations of the solar environment and heliosphere. Both STEREO spacecraft are equipped with an EUV imager that observes the Sun in four EUV wavelengths. The 195 Å passband used here has a characteristic temperature of $\approx 1.6$ MK and images the upper chromosphere and hot plasma with a spatial resolution of $3.5^{\prime \prime }$.

A movie detailing the evolution of the filament in EUV as seen by SDO and STEREO-B is included in the online version of this article as an Electronic Supplementary Material; it is associated with Figure 2. Because of an additional (unrelated) simultaneous eruption in the northern hemisphere but behind the limb, it was not possible to disentangle the two co-temporal CMEs in images from the *Large Angle Spectrometric Coronagraph* (LASCO: Brueckner *et al.*, 1995) instrument onboard the *Solar and Heliospheric Observatory* (SOHO: Domingo, Fleck, and Poland, 1995).

## Results

### Evolution of the Magnetic Flux in the Filament Channel

**Figure 3:**
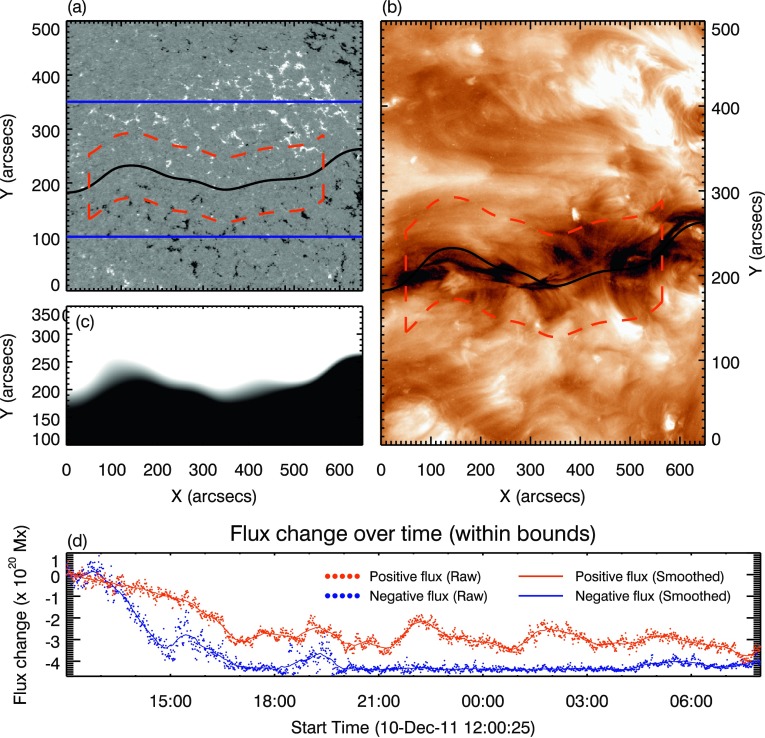
Magnetic-field evolution within the filament channel. Panel **a**: HMI LOS magnetogram rotated to disc centre. The *solid-blue lines* show the bounds of the LOS magnetogram smoothed to define the position of the PIL as in **c**, the *solid-black line* shows the position of the PIL based on the smoothing regime, the *dashed-red lines* define the bounds of summation defined by features associated with the filament observed in the AIA 193 Å and 171 Å passbands, as in **b**. Panel **b**: AIA 193 Å passband image corresponding to the same FOV as the HMI image in **a**, used to define the region enclosed by the *red-dashed line*. The image has been saturated to emphasise filament material. Panel **c**: Result of the smoothed HMI LOS magnetogram that defines the location of the PIL. Panel **d**: The evolution of the flux contained within the boundaries defined in **b**. Cancellation is present within the specified bounds until $\approx 18{:}00$ UT, after which the trend plateaus and remains nearly constant.

Tracking the evolution of magnetic flux along the filament channel can give an insight into the evolution of the magnetic environment around the filament of interest. This can be done by isolating the area that the filament channel occupies in HMI data. To do this, the HMI region of interest (ROI) was selected to include the estimated location of the PIL of the filament and a small amount of the positive and negative region either side. The image was then successively smoothed by 1000 iterations of a $4.5^{\prime \prime }$-width window to reduce the noise in the ROI. This approach was taken because restricting the saturation to $\pm 1$ G was found to produce an image with a large noise component, making it impossible to obtain a location for the PIL. The iterative approach results in an image with a clear neutral line indicating a PIL, as shown in Figure 3b, despite a very diffuse PIL in the LOS data. This was visually checked against the location of the filament in the SDO/AIA 193 Å and 171 Å passbands. The width of the region used to sum the magnetic flux was then set by identifying features connected to the filament channel using the 193 Å and 171 Å passbands. The upper bound was set to include the western footpoint and exclude the small cancelling bipole present at $\approx (390^{\prime \prime }, 270^{\prime \prime })$ in Figure 3, and the lower bound was set at the latitude where the large mass deposit was seen to impact the surface. The east and west boundaries were defined by the appearance of the filament material. This ensures that as much as possible of the flux contained within the filament channel was included within the summation limits. However, this does not account for the inclusion of additional flux that might be unrelated to the filament. Similarly, the boundary selection is a “by-eye” process, and assumes that the features chosen accurately represent the boundary of the material to be studied. A threshold of $\pm 30$ G was applied to the ROI summation to reduce the noise in the result. This approach was then applied to all of the corrected magnetic data throughout the observation period from 12:00 UT to 08:00 UT on 10 and 11 December 2011. This produces a value of summed positive and negative flux within the specified bounds for each time step. The results for the positive and negative flux variation were then smoothed over time using a 50-point average to subdue the small-scale variation.

Figure 3d shows a large decrease in flux (interpreted as cancellation) present within the specified bounds at the beginning of the observation period. This decrease corresponds to a value of $\approx 3.5\times 10^{20}$ Mx of unsigned flux. The flux cancellation along the PIL is then seen to plateau after $\approx 18{:}00$ UT on 10 December 2011 and remains nearly constant for the rest of the observation period up to and after the eruption.

### Bipole Emergence

Bright, low-lying loops observed in EUV close to the edge of the filament channel were seen to form several hours *prior* to the eruption on 11 December 2011 at 05:53 UT. The corresponding photospheric signature of this flux emergence was identified in LOS magnetograms as opposite-polarity signatures growing and separating (forming a bipole) within the positive region of the diffuse bipolar region hosting the filament. The orientation of the bipole, observed using HMI, was such that its negative polarity was closest to the PIL of the region. Such adjacent, oppositely signed flux is a configuration established to be favourable for reconnection between the two systems (Feynman and Martin, 1995).

**Figure 4:**
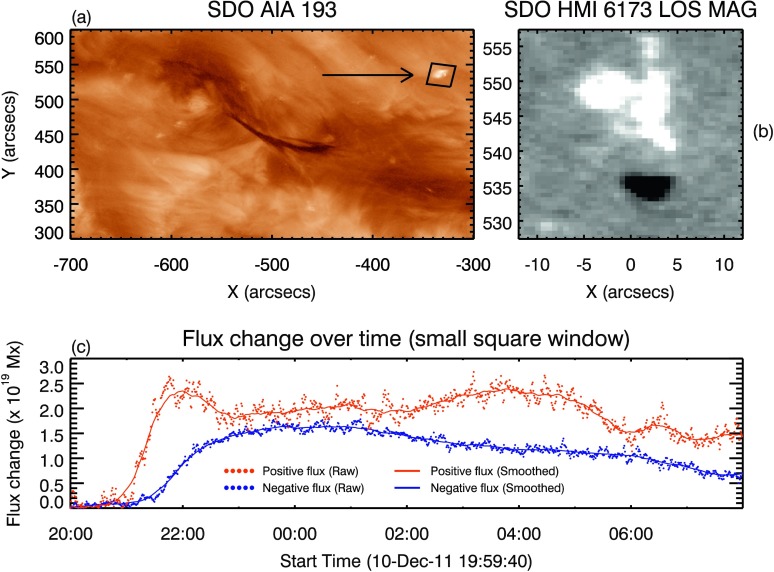
Evolution of a small-scale emerging flux region at the edge of the filament channel. Panel **a**: Location of the bipole emergence with respect to the filament of interest, as indicated by the *arrow* and *black box*. Panel **b**: The ROI, corresponding to the *black box* in **a**, used for flux summation of the bipole emergence (positive = *white*, negative = *black*). Note that the bipole is surrounded by a positive-polarity magnetic environment. Panel **c**: The evolution of the positive and negative flux contained within the small box surrounding the emerging bipole seen to the north-west of the filament. Emergence begins at approximately 21:00 UT on 10 December 2011, and negative flux, associated with the emergence only, peaks at 00:30 UT on 11 December 2011. Both AIA and HMI images have time stamps of 00:41 UT.

Figure 4 shows the evolution of the flux contained within a small box surrounding the region into which the bipole emerged. The region contained within the FOV was located at $-32^{\circ} $ longitude and $+33^{\circ} $ latitude from Sun centre at 21:00 UT. The prepared data were de-radialised and de-rotated to allow the radial component of the magnetic field to be estimated, and to rotate the ROI to disc centre, respectively. This placed the de-rotated region of interest at $0^{\circ} $ longitude and $+33^{\circ} $ latitude. The ROI was then restricted to $\pm 12^{\prime \prime }$ in $x$ and $528\,\text{--}\,557^{\prime \prime }$ in $y$, as seen in Figure 4b, and the sum total of the LOS magnetic fields with $|B|>30$ G within the enclosed area were calculated, with this process repeated for all time steps. The result was then smoothed by a moving 50-point average to subdue the small-scale variations and isolate just the overall trend. At 20:00 UT, in Figure 4, the values of the positive and negative flux were set to zero to isolate the emergence of the bipole.

The emergence began at approximately 21:00 UT on 10 December 2011, indicated by the increase in both positive and negative flux in Figure 4. It is noticeable that the negative flux increased at a slower rate than the positive flux. Owing to the predominance of positive polarity in the region of emergence, this lag may be due to the emerging negative flux interacting almost immediately with its surroundings and either remaining below the $\pm 30$ G threshold or cancelling entirely. Alternatively, it could be an artefact of the simple assumption used in the calculation of the radial component, therefore introducing a flux imbalance in the photospheric field. It is also clear that the evolutions of the positive flux and negative flux were non-identical. The ROI into which the bipole emerges was sufficiently isolated with respect to nearby flux that no flux breached the boundary of the ROI during the observation period. Despite this, the positive component displays sporadic variations, suggesting that pre-existing polarity within the $\pm 30$ G threshold was breaching and receding through this threshold over time. This indicates that the trend shown by the negative flux is more representative of the emergence of just the bipole into the ROI, as its flux evolution only corresponds to that of the emergence. The negative flux can be seen to peak at approximately 00:30 UT on 11 December 2011 with a value of $\approx 1.6\times 10^{19}$ Mx of unsigned flux emergence recorded.

### Morphological Analysis of Flows

**Figure 5:**
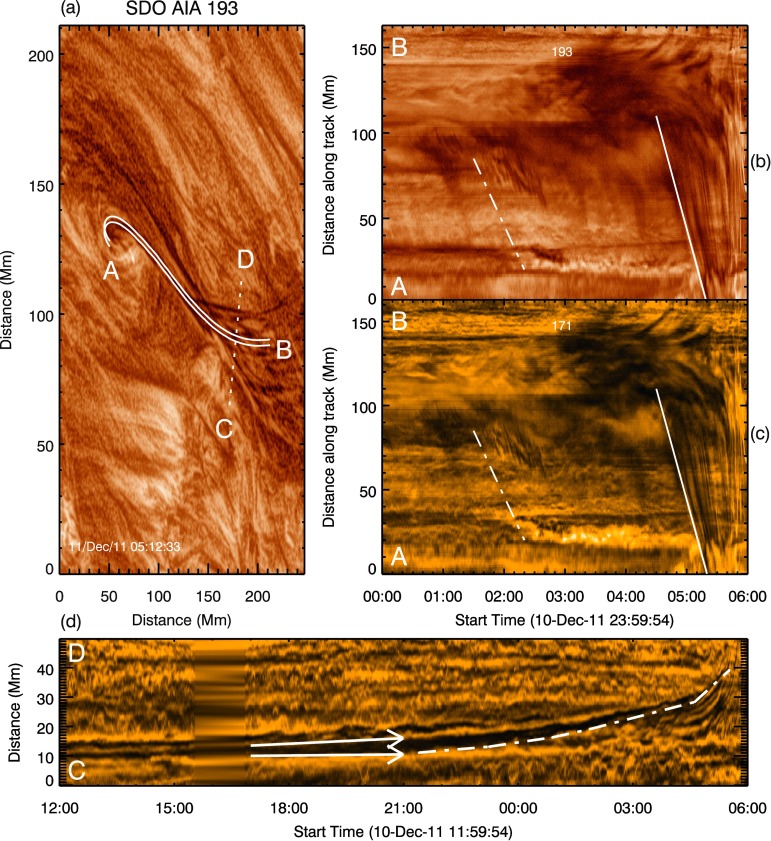
Tracking intensity variations along and perpendicular to the filament axis. Panel **a**: The de-rotated ROI used to specify the vectors that trace the motions of interest. The region contained within the *white-solid lines* (*A*–*B*) was used to construct the stack-plots shown in panels **b** and **c**. The *dotted-white line* (*C*–*D*) indicates the location of the line used to construct the stack-plot shown in panel **d**. Panel **b**: The temporal variation in pixel intensity, averaged across the width of separation of the two lines, along the axis of the flows over time for the 193 Å passband. Panel **c**: The same as **b**, but for the 171 Å passband. *Lines* indicate start of the initial and the large mass-unloading. Panel **d**: The temporal variation of the pixel intensity along the *dotted-white line* (**C**–**D**) in **a**. Passbands were processed using the MGN technique to isolate the fine structure of the flows.

Flows are seen by SDO/AIA to propagate away from the apex of the dynamic portion of the filament just *prior* to the eruption. As the flows propagated away from the apex, they appear to travel along a previously unidentified path that led away from the filament. We define this new path as the axis of the flows. Although the flows are seen as dark “blobs”, images taken using SDO/AIA are sensitive to temperature variations in the emissive source. Thus any intensity variations observed could be temperature variations of the observed plasma environment or physical density variations and therefore plane-of-sky motions of the plasma itself. In order to distinguish the nature of the intensity variations as flows, the motions need to be temperature independent. By plotting the intensity of pixels along a vector against time, it is possible to isolate how these intensity variations are evolving along the given vector; we refer to these as stack-plots. The stack-plots shown in Figures 5b and c detail the evolution of intensity variations recorded along the static white-solid vector specified in Figure 5a. The location of the vector was specified by hand-clicking along the path that the largest intensity variation is seen to have taken from the apex of the filament to the eastern footpoint just *prior* to the partial eruption of the filament. From the initial hand-clicked vector, a width was specified of two pixels [870 kilometres] either side to allow an average to be taken across the separation. This average was introduced to reduce noise in the recorded value of pixel intensity and to increase the signal of the potential flows against the background of the solar surface. Figures 5b and c show the temporal variation in intensity along the path to the apex from the eastern footpoint of the filament for the 193 Å and 171 Å passbands, respectively.

The first large intensity variation observed to travel from the apex of the filament to its eastern footpoint occurred at $\approx 01{:}30$ UT. This is seen in Figures 5b and c as a darker feature originating at $\approx 85$ Mm along the track and impacting the surface at 02:30 UT, as indicated by the brightenings at $\approx 30$ Mm. As the vector was specified along the path taken by the largest intensity variation just *prior* to eruption, and given that filaments are highly dynamic structures, it does not fully trace the path of all intensity variations. It is however appropriately placed to record the initial movement from the apex, and the final movement and impact of the intensity variations at the surface. The surface brightenings are present throughout the lead-up to the eruption, between 20 and 30 Mm from 02:30 to $\approx 05{:}00$ UT in Figure 5, suggesting the process causing the intensity variations decreased in extent but did not cease. The intensity variations were then seen to darken and dramatically expand from the filament centre to the surface, approximately one hour before eruption, noted in Figures 5b and c by the grouping of linear streaks angled towards 0 Mm, *i.e.* the eastern footpoint. This larger motion then continued throughout the final hour leading up to the eruption. Intensity variations were also observed to propagate from the apex of the filament to its western footpoint, but they were far less intense or dynamic. These intensity variations on the western side remained constant after their initiation at 02:50 UT, persisting until the eruption of the filament, and simply served to highlight the location and large size of the western footpoint.

In addition to the stack-plots made from the vector A–B in Figure 5, the second vector (C–D) is a perpendicular bisector of the length of the filament. The change in the orientation of the dynamic portion of the filament over time is presented in Figure 5d. Between $\approx 17{:}00$ and 21:00 UT on 10 December, the filament can be seen to widen, as highlighted by the arrows. After the expansion of the filament, the entire filament appears to undergo a bulk anti-clockwise rotation that persists up to the partial eruption of the filament at $\approx 05{:}53$ UT on 11 December.

Figure 5 shows that the observed flows were spatially co-located in both the 171 Å (0.63 MK) and 193 Å (1.3 – 2 MK) passbands. This indicates that the variations were not temperature sensitive, but were physical density variations showing the motions of material within the filament. As these intensity variations were indeed material motions, we can now consider the density of these flows with respect to the density of the rest of the filament structure.

### Density Evolution

Cool, dense chromospheric material that is suspended in the hot, tenuous corona (*i.e.* filament material) appears in absorption in extreme ultraviolet (EUV) wavelengths below the Lyman continuum limit at 912 Å; photons are removed from the LOS predominantly by photoionisation (Williams, Baker, and van Driel-Gesztelyi, 2013), so the efficiency of this removal is a function of wavelength. The temperature of this material, however, is low enough to assume that only negligible emission occurs at these wavelengths (Landi and Reale, 2013). In this case, the optical depth of the material [$\tau $] is defined by the column number density [$N$] multiplied by the cross-sectional area of photoionisation [$\sigma $],

1 $$ \tau ~=~N~\sigma (\lambda ) , $$

which will reduce the intensity of radiation passing through the material as

2 $$ I_{\mathrm{obs}}=I_{\mathrm{b}}\exp {(-\tau )} , $$

where $I_{\mathrm{obs}}$ is the final observed intensity and $I_{\mathrm{b}}$ is the intensity before passing through the material (“background”). The cross-sectional area of hydrogen and both neutral and singly-ionised helium is very similar at wavelengths below 227 Å when weighted by the solar chemical abundances given by Grevesse, Asplund, and Sauval (2007) ($A_{ \mathrm{H}}=1$, $A_{\mathrm{He}}=0.085$), allowing the column number density of hydrogen to be calculated from the total optical depth,

3 $$ N_{\mathrm{H}}~\geq ~\frac{\tau_{\mathrm{tot}}}{2A_{\mathrm{He}} \sigma_{\mathrm{HeII}}} $$

(see Williams, Baker, and van Driel-Gesztelyi, 2013, for a rigorous derivation.)

The total optical depth of such material may be estimated provided the “background”, or rather the unattenuated radiation field,^1^ can be reasonably approximated. This may be done for highly dynamic material by taking an image co-spatial to the examined material some moments in time before or after the material is in that particular FOV. For less dynamic material, the background could be estimated from surrounding areas that are unobscured by cool, dense material. Therefore, provided two suitable images exist (one of the material to be measured, and one to estimate the unattenuated field), a lower limit on the hydrogen column number density may be calculated.

#### Polychromatic Method

As previously mentioned, if the filament material were observed in ≥ three wavelengths below 227 Å (the ionisation limit for He ii), the optical depth could be used to constrain a model that includes the fraction of emission; the unattenuated radiation field includes not only background radiation, but also emission from hot coronal material between the filament material and observer. Furthermore, fine structuring in the filament material may allow background emission to pass through unobstructed, and as such a pixel-filling factor should be considered. Therefore, the intensity observed is given by

4 $$ I_{\mathrm{obs}}=I_{\mathrm{b}}\bigl(f\exp {(-\tau )}+(1-f)\bigr)+I_{ \mathrm{f}} , $$

where $f$ is the pixel-filling factor (*i.e.* the fraction of each pixel occupied by material) and $I_{\mathrm{f}}$ is the foreground emission. Rearranging, we have

5 $$ 1-\frac{I_{\mathrm{obs}}}{I_{\mathrm{b}}+I_{\mathrm{f}}}=f\frac{I _{\mathrm{b}}}{I_{\mathrm{b}}+I_{\mathrm{f}}}\bigl(1-\exp {(-\tau )}\bigr) , $$

where the unattenuated radiation field is approximately equal to $I_{\mathrm{b}}+I_{\mathrm{f}}$ (emissivity blocking, the emission that would be emanating from the hot corona in the location of the filamentary material were it absent is negligible due to the small volume relative to the rest of the corona), and so the left-hand side of Equation 5 is measurable, denoted as $d(\lambda )$. On the right-hand side, a substitution can be made,

6 $$ f\frac{I_{\mathrm{b}}}{I_{\mathrm{b}}+I_{\mathrm{f}}}=G. $$

This reduces the model to have two free parameters, and so if $d$ is measured in three or more wavelengths, the model may be constrained by, *e.g.*, a least-squares fit. Although the multiple wavelengths below 227 Å required by this technique are captured by SDO/AIA, from this point of view (POV), the unattenuated radiation field is not only more dynamic but also more structured. This introduces uncertainties in the estimated radiation field and hence the calculated density. For more detail on this method, see Heinzel *et al.* (2008), Williams, Baker, and van Driel-Gesztelyi (2013), and Carlyle *et al.* (2014).

**Figure 6:**
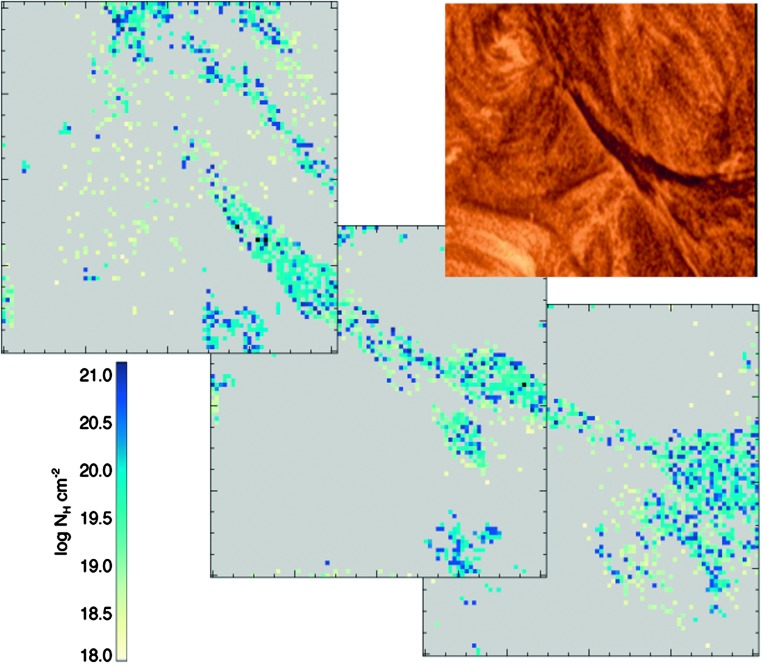
Mosaic of column number density measurements of the dynamic portion of the filament from SDO/AIA captured at 01:51 UT. The mosaics have a $48^{\prime \prime }\times 48^{\prime \prime }$ FOV, with each tick separated by $3^{\prime \prime }$. *Top-right*: Intensity image from SDO/AIA 193 Å with the filament as seen at 01:51 UT.

In Figure 6 we study the density of the dynamic portion of the filament. A mosaic is presented, as computing the column number density for a square of this size would be much more computationally demanding, and given that the corners are of no interest, this was deemed unnecessary. Furthermore, the unattenuated estimation assumption is more difficult to satisfy, as the Sun is highly dynamic and structured, so using three similar, smaller frames increases the reliability of the results. The requirement for an unattenuated background field was well satisfied at 01:51 UT, which is why this time was chosen for the analysis.

**Figure 7:**
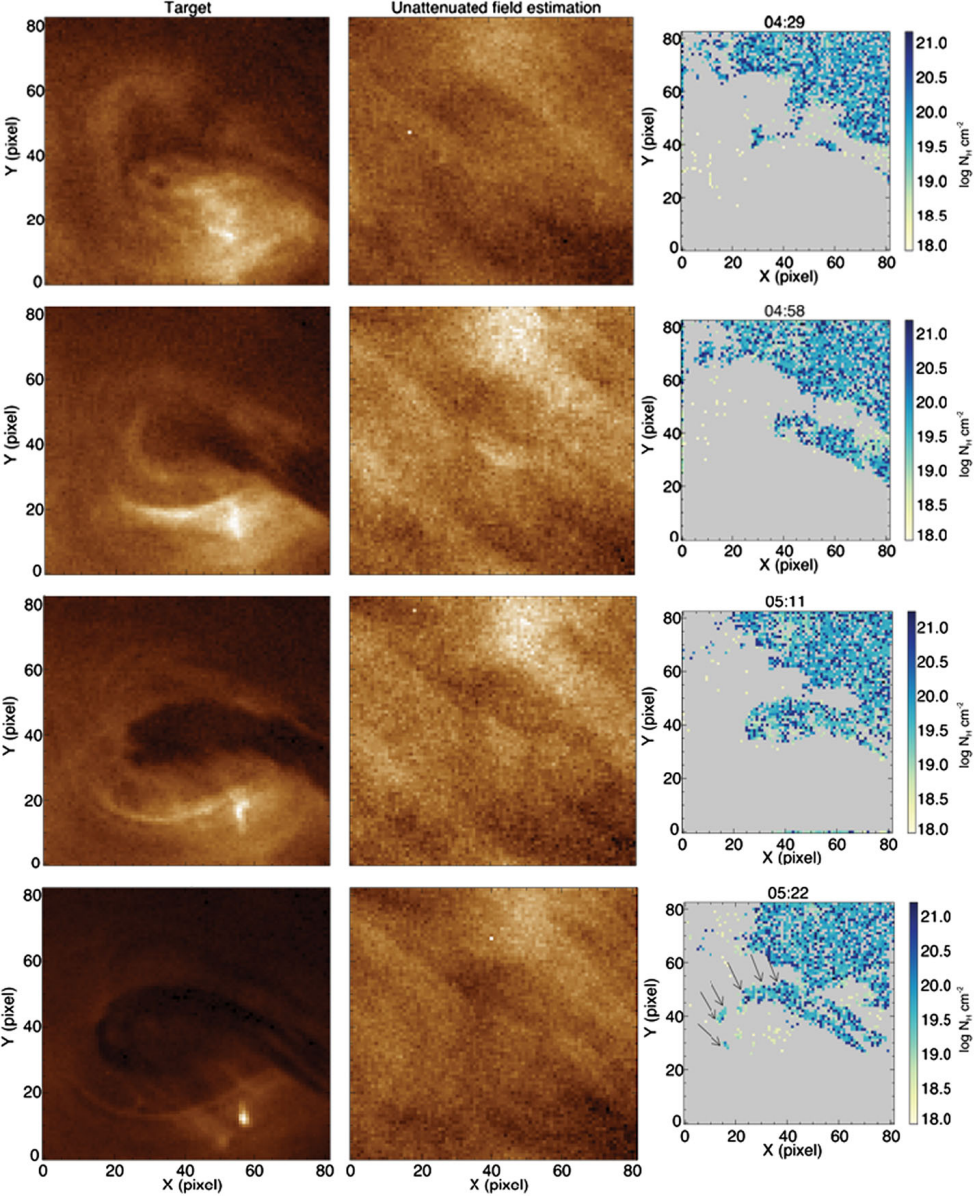
Density evolution of the large mass-unloading between 04:29 and 05:22 UT on 11 December 2011, using the polychromatic method applied to SDO/AIA data. *Left*: FOVs used in the density-determination method that include the footpoint of the large mass-unloading defined in Figure 1a. *Middle*: Unattenuated radiation-field estimation for the intensity image shown in the *left*. *Right*: Result of the density-determination method based on the specified target and unattenuated field estimate. The hooked shape of the dense object at 05:22 UT, as indicated by the *arrows*, is due to the material approaching the surface *via* curved field lines. All times indicated above the panels are in UT. The spatial scale is $\approx 0.6^{\prime \prime }$ *per* pixel.

In Figure 7 we focus on the motion of the large density flow from the filament apex to the eastern footpoint. As before, a sufficiently small FOV was chosen, and the result of applying the density-determination method to this FOV at multiple times is presented, revealing the evolution of density in the frame throughout the mass-unloading. Material is clearly seen to enter the FOV from the apex of the filament in the centre-right of each image and follow a curved path corresponding to the curved field lines from the filament apex to the surface.

The polychromatic technique applied to both the entire dynamic portion of the filament, and the large mass-unloading as seen in the SDO/AIA data returns a mean column number density of roughly $1\times 10^{20}$ cm^−2^.

#### Monochromatic Method

**Figure 8:**
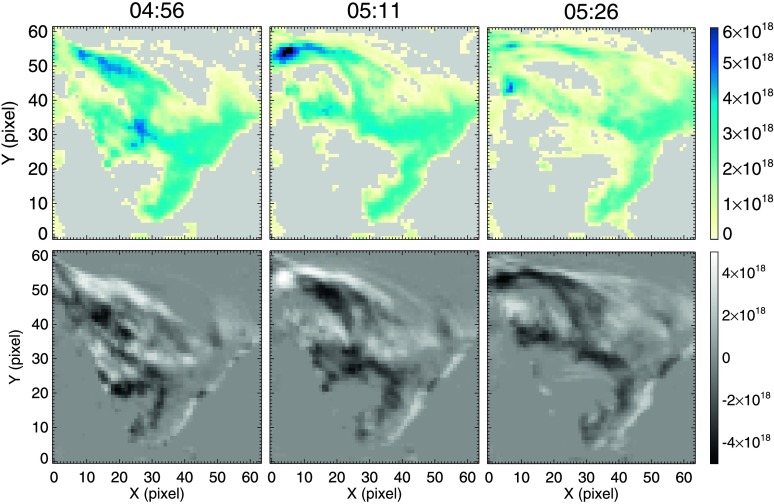
Evolution of the column number density and flows in the dynamic portion of the filament as seen from STEREO-B/EUVI. *Top row*: Evolution of the column number density, measured in $\mathrm{N}_{ \mathrm{H}}~\text{cm}^{-2}$, detailing the mass-unloading initiated at $\approx 04{:}40$ UT on 11 December 2011. The large mass deposit can be seen as an increase in column number density at the *top-left of the image*. *Bottom row*: Running-difference images of the column number-density evolution measured in $\Delta \mathrm{N}_{\mathrm{H}}~\text{cm}^{-2}$. Features becoming more (less) dense in time appear *white* (*black*). This further highlights that the mass is moving towards the north-east footpoint of the filament (*top-left of each image*). The spatial scale is $\approx 1.6^{\prime \prime }$ *per* pixel.

On 11 December 2011, STEREO-B was ideally positioned to view the erupting filament at a near perpendicular LOS with an angle $\theta_{\text{STEREO-B}}=108^{\circ} $ to the Sun–Earth line. This provided a rare opportunity to view an eruption contemporaneously both on-disc (SDO/AIA) and off-limb (STEREO-B/EUVI), and help disentangle the structure of the filament that would be otherwise unachievable with single-perspective observations. From the POV of STEREO-B, the filament was projected against a slowly changing background of the corona, and as such, the unattenuated background radiation field was well approximated by using an exposure taken in the location of the filament following its eruption. This method was applied only to data collected by the 195 Å passband onboard STEREO-B/EUVI as the cadence for the 171 Å channel was too low to be useful here. Because we only have access to a single wavelength for these observations from STEREO-B and therefore cannot estimate a filling factor or fraction of emission, a density determination is only possible using the simpler model of Equation 3, a monochromatic estimation. Unfortunately, not being able to include the filling factor or fraction of emission in the estimation results in a lower estimation of the column number density. The results of this analysis are summarised in Figure 8.

**Figure 9:**
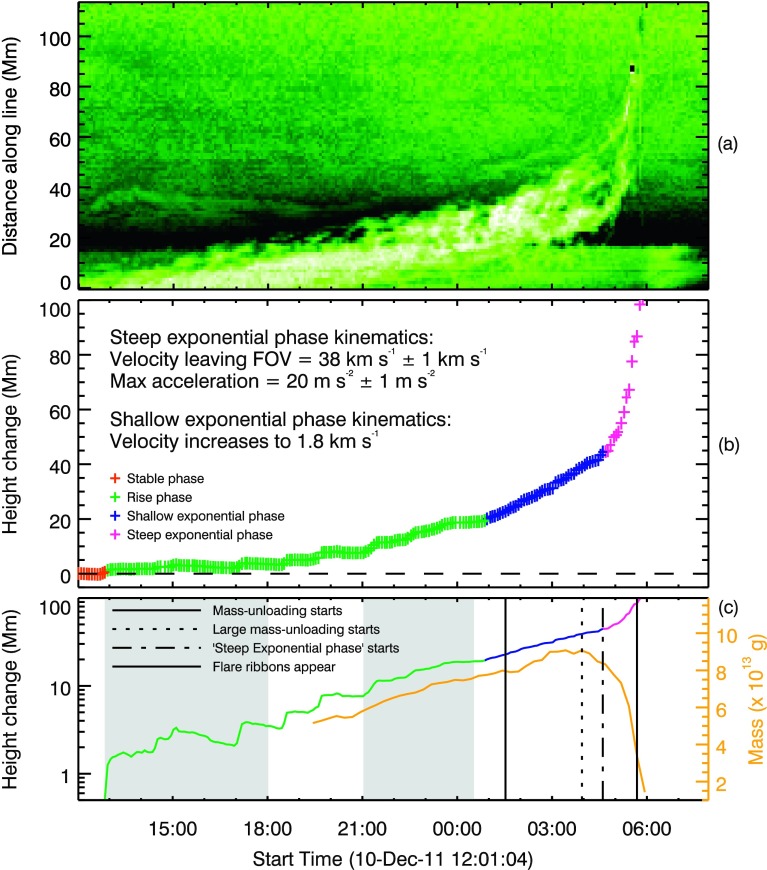
Evolution of the filament height and mass with time. Panel **a**: The height–time stack-plot taken along the line specified in Figure 1c using a reversed colour table. Panel **b**: The solar-rotation-removed evolution of the dynamic portion of the filament as it evolved and erupted. *Red* indicates the stable phase, *green* indicates the rise phase, *blue* indicates the shallow-exponential phase, and *magenta* indicates the steep-exponential expansion phase. Kinematics are derived from the fitting of an exponential function to the *magenta region* of evolution, and a line to the *blue region*. Panel **c**: The *h–t* profile as in **b**, but with a logarithmic scaling, compared to the evolution of mass contained within the FOV enclosing the filament over time, as seen by STEREO-B/EUVI. The initial increase in mass corresponds to larger portions of the filament being visible above the limb over time due to solar rotation, and can therefore be considered to be artificial. *Shaded patches* indicate the times of flux cancellation recorded along the PIL and the nearby bipole emergence. *Vertical lines* indicate (from left to right) the time of first visible mass-unloading, the time of the largest mass deposit, the start of the steep-exponential expansion, and the initial appearance of the flare ribbons and twin EUV dimmings.

The monochromatic technique applied to the STEREO-B/EUVI data returns an average lower column-number-density limit of approximately $4.5\times 10^{18}$ cm^−2^ for the filament, as seen in the top panels of Figure 8. As before, applying this method to many snapshots over the course of the evolution of the filament highlights the evolution in column number density over time. These results show a gradual increase in total mass in the target frame from $4\times 10^{13}$ g to $8.8\times 10^{13}$ g over approximately eight hours, consistent with the filament slowly rising into the FOV; the evolution of mass is shown Figure 9c. This suggests that the filament rising into the FOV and associated increase in measured mass has partially masked the initiation of the smaller-scale mass-unloading at 01:30 UT. This increase is followed by overdensities moving in the direction of the north-east footpoints (highlighted by subtracting each column-number-density map from the next, creating a “running-density difference” image as shown in the bottom panels of Figure 8), before these overdensities appear to suddenly drain down towards the eastern footpoint, reducing the total target mass to $2.2\times 10^{13}$ g in just over an hour, as seen in Figure 9c.

These results indicate that the filament studied here was large and dense throughout the lead-up to its eruption. A large and sudden decrease in the density of the material contained within the filament is apparent from 04:00 UT onwards to eruption, decreasing the total mass in its measurable portion by more than two thirds ($\approx 6.6\times 10^{13}$ g).

### Filament and Plasma Kinematics

In the previous sections we have identified several large, dense pockets of material moving away from the apex of the filament structure and down to the surface. The 1D velocity estimate, derived from the stack-plots in Figure 5, of the large mass-deposit leaving the apex of the filament is $\approx 28$ km s^−1^. The second perspective offered by STEREO-B/EUVI shown in Figure 8 indicates that the large mass deposit originated from a height of $\approx 40$ Mm and $\approx 30$ minutes earlier than suggested in the stack-plots of Figure 5. The combination of the stack-plot observations made from both spacecraft therefore suggests that the mass-deposit had a 2D linear acceleration of $\approx 12$ m s^−2^, reaching a velocity of $\approx 31$ km s^−1^ as the material approached the surface. The acceleration of the unloading mass is an order of magnitude lower than free-fall at the solar surface.

As previously stated in Section 2, the dynamic portion of the filament erupts whilst the restrained portion does not. Panel a of Figure 9 shows the height–time (*h–t*) evolution of the dynamic portion of the filament from the perspective of STEREO-B. The evolution of the filament described in the stack-plot is directly in the plane of sky, the eruption occurred at $\approx 90^{\circ} $ to the Sun–STEREO-B line and is therefore assumed to be approximately radial. The evolution of the filament height with time was measured by selecting the leading edge of the filament by hand. This was repeated six times to minimise user bias and provide an average position. The contribution to the *h–t* evolution due to solar rotation was then removed, revealing four main evolution domains within the lead-up to the eruption; these are summarised in Figure 9b:

i) A stable phase (red) in which it appears that the only contribution to the stack-plot evolution was due to solar rotation. The average position of this phase is set as a distance of zero so that any subsequent *h–t* evolution refers to a deviation from the height of the filament during this stable phase.
ii) A rise phase (green) that describes the deviation from stable phase and includes the initial lift-off from the surface.
iii) A *shallow* exponential phase (blue), defined as the *h–t* evolution, is approximately linear when plotted on a logarithmic scale, as in Figure 9c.
iv) A *steep* exponential phase (magenta). Fitting the exponential phase of the *h–t* profile with the exponential function suggests that the final radial velocity of the filament leaving the FOV was $v \approx 38\pm 1~\text{km}\,\text{s}^{-1}$, a higher-than-average velocity for a quiescent filament eruption according to Loboda and Bogachev (2015). An acceleration of $a\approx 20\pm 1~\text{m}\,\text{s}^{-2}$ was found to follow the reduction in mass within the filament and is indicative of the initiation of the impulsive acceleration phase of a CME (Schrijver *et al.*, 2008).

Figure 9c compares the *h–t* profile of the filament using a logarithmic scale with the evolution of mass from the column-number-density measurements of Section 4.4. It is shown that the expansion of the filament between $\approx 01{:}00$ UT and 04:40 UT is exponential, as indicated by the linear evolution. The unloading of mass is seen to begin at $\approx 04{:}00$ UT; after a large portion of the mass had drained from the system, the *h–t* profile is seen to have accelerated to a larger exponential expansion.

## Discussion

Filament (prominence) eruptions, a progenitor of CMEs, have been studied for many decades (Parenti, 2014). The myriad studies describing the magnetic destabilisation of a solar filament have greatly advanced our understanding of the different solar eruption triggers and drivers (Aulanier *et al.*, 2010). However, whilst the role played by the material contained within the flux rope has been acknowledged by several authors (*e.g.* Fan, 2017), it is largely neglected, with the general consensus being that the material is unimportant (*e.g.* Török and Kliem, 2005; Fan and Gibson, 2007; Török *et al.*, 2011). Here we present an event that strongly indicates that the internal dynamics of the mass cannot be ignored when considering an evolving and destabilising flux rope.

At the start of the period chosen for analysis of this event (12:00 UT on 10 December), a decrease in surface magnetic flux was recorded along the PIL that lay underneath the filament of interest. This indicated flux cancellation and an increase in concentration of the non-potentiality along the PIL. If there were a pre-existing flux rope along the PIL, the negative trend of the total flux indeed implies that flux was added to the flux-rope system. The easternmost border of the summation bound in Figure 3 lies at $\approx 60^{\circ} $. Because of the increased noise in the LOS magnetic data past the $60^{\circ} $ limit (Hoeksema *et al.*, 2014), we therefore cannot study the evolution in flux further back in time and can only infer the connection to the flux-rope formation process *via* flux cancellation described by van Ballegooijen and Martens (1989). The plateauing trend in the flux evolution suggests that the reconnection driving the formation of the proposed flux rope had ceased by, or just after, 18:00 UT on 10 December. This non-potential magnetic system would find a new equilibrium; for a flux rope, this can be achieved through expansion and an associated increase in height. It is possible that the widening of the filament (Figure 5d) is the observational signature of the filament rising towards the observer. Because of the data gap in the AIA 171 passband between 15:32 and 16:50 UT on 10 December, it is unfortunately not possible to extrapolate this backwards in time.

The emergence of the nearby bipole studied in Section 4.2 began at $\approx 21{:}00$ UT, after the plateauing of flux cancellation recorded along the PIL below the filament of interest. Work by, *e.g.*, Feynman and Martin (1995) and Chen and Shibata (2000) suggested that this emerging bipole was preferentially oriented for reconnection with the field overlying the filament of interest, *i.e.* the negative polarity of the bipole was closer to the negative polarity of the hosting bipolar field. If there had been an interaction between the two systems, then the overlying field above the filament would have been weakened as a result of the proposed reconnection, as described in Williams *et al.* (2005). The filament would then have been able to expand to a greater height within the corona at a speed proportional to the reconnection rate between the bipole and the field overlying the filament. Based on the stack-plot presented in Figure 9a, panels b and c of the same figure show that the filament appeared to have been in equilibrium for approximately an hour *prior* to 21:00 UT. After 21:00 UT and the beginning of the emergence of the bipole, the same plots describe the filament as having resumed its expansion through the corona. The *h–t* evolution of the filament is then seen to have plateaued at 23:30 UT. At this time, the nearby bipole was approaching the peak of its emergence (see Figure 4, actual peak at 00:30 UT on 11 December), and therefore the reconnection rate between the two systems would have slowed and ultimately ceased altogether. It is worth noting that an additional, smaller bipole is seen to have emerged at $\approx 20{:}30$ UT beneath the western portion of the filament. Observationally, it appears that the field topology in the vicinity of this flux emergence also re-organised to some extent, as indicated by the sporadic bright extensions away from the location of the bipole. However, the orientation of this smaller bipole with respect to the surrounding field suggests that it was not favourably oriented for reconnection, as was the case for the larger bipole that has been studied in Section 4.2. Nevertheless, it is possible that this smaller bipole was involved in the evolution of the system, even if to an unmeasurable degree. Whilst the presented analyses suggest a tenuous connection between the studied emergence and the evolution of the filament, the post-eruption large twin EUV dimmings associated with the footpoints of the proposed flux rope (*cf.* James *et al.*, 2017) are seen to have migrated counter-clockwise, and the western dimming is seen to have approached and eventually enveloped the location of the studied bipole, indicating a relationship between the bipole and the footpoints of the erupting CME. However, it is unlikely that such small bipoles were the sole influence on the evolution of the filament height after 21:00 UT on 10 December.

EUV and LOS magnetogram observations of the region surrounding the filament of interest suggest that the proposed flux rope containing the filament would have been left-handed with a negative helicity, as inferred by its location in the northern hemisphere, its roots in a positive-leading and negative-trailing diffuse bipolar region (Figure 1b), and the shear angle of the associated loops. Green *et al.* (2007) suggested that a flux rope in this configuration should rotate anti-clockwise about its PIL as it expands. Figure 5d is a stack, plot defined by a vector that is a perpendicular bisector of the axis of the dynamic portion of the filament. In this plot, at $\approx 21{:}00$ UT on 10 December, the filament is seen to have started rotating counter-clockwise about its PIL. The combination of this observation and the previously discussed expansion of the filament beginning at approximately the same time is consistent with the conclusions presented by Green *et al.* (2007). In addition to this, the counter-clockwise rotation can be seen to continue throughout the period leading up to the eruption at 05:53 UT on 11 December, whereas the emergence of the bipole – the possible cause of the height increase from 21:00 UT on 10 December – ceased by 00:30 UT on 11 December. This suggests that the observed persistence in rotation is in fact due to the proposed flux rope becoming kink-unstable. Nevertheless, this does not necessarily override the role of the emerging bipole in the expansion of the filament, as the combination of the two mechanisms is likely to have influenced the temporal evolution of the height of the studied filament.

We have so far referred to the magnetic structure that possibly contains the filament as having a flux-rope topology. However, the event described here lacks certain features that are usually identified in observations when a flux rope is in fact present; for example, there is little evidence of a cavity in SDO/AIA 193 Å images that would outline the shape of a possible flux rope when passing the eastern limb. The only explanation for this is that the angle that the filament makes with the LOS of either the SDO or STEREO-B is not optimal for a cavity observation, as we are not observing the structure along its axis (*cf.* Gibson *et al.*, 2006; Forland *et al.*, 2013). However, the combined observations of flux cancellation in the filament channel, the suggested onset of kinking, the post-eruption twin EUV dimmings, and the brightenings along the length of the filament during the eruption that appear to outline helical field, most visibly seen in the movie associated with Figure 2, are most consistent with the flux-rope theory.

Interestingly, the filament can be seen to have fanned out during this kinking. This is most evident on the western side of the filament as the field lines associated with the fanning are highlighted by material suspended along their length, as previously stated in Section 4.3. In the movie associated with Figure 2, it is possible to see that the flows trace the curvilinear paths of the fanning field lines from $\approx 01{:}30$ UT until eruption. As we have assumed a flux-rope configuration of the magnetic environment surrounding and suspending the filamentary material, by studying the magnetic topology of simulations by authors such as Roussev *et al.* (2003), Mei *et al.* (2017), and Guo *et al.* (2017), it is difficult to reconcile how the highly twisted field lines of these studied flux ropes could produce the observed linear-like motions of plasma; plasma-$\upbeta $ (plasma pressure/magnetic pressure) is low in the corona and therefore charged material is line-tied. Interestingly, extrapolations of less strongly twisted flux ropes, such as those by Su *et al.* (2011) and Jiang *et al.* (2014), are easier to compare to the observations as they contain very weakly twisted field lines that pass through the axes of the extrapolated flux ropes. However, as pointed out by Aulanier and Démoulin (1998), the location of the dips that contain the cool, dense material of the filament are unlikely to reach entirely up to the axis of the flux rope, with the majority of the material lying in the lower portions. Nevertheless, the assumption that the filament material lies entirely within the dips of the magnetic flux rope is a first-order approximation. With the inclusion of the thermodynamic instability, which is thought to occur in the solar environment (Field, 1965), material might be suspended in higher portions of a flux rope, including the more weakly twisted field of the flux-rope axis. In addition, work completed by Su *et al.* (2011), and more recently by Polito *et al.* (2017), was able to successfully reconstruct the diffuse footpoints of flux ropes, as their observations had initially indicated, a feature not currently achievable through simulations. Therefore the combination of magnetic dips, the thermal instability, and the possibility of a diffuse flux-rope footpoint offer explanations to the observations presented in this article, *i.e.* that the material can be seen to travel along curvilinear field lines associated with the flux rope and are rooted in regions that cross supergranular boundaries.

Continuing with the evolution of the filament, Figure 9 shows that the proposed flux rope containing the filament became marginally unstable at 01:00 UT on 11 December, as denoted by the linear height–time evolution on the logarithmic scale in Figure 9c. As a consequence of flux-rope expansion, flux-rope field lines that suspend filament material above the surface increase in their gradient with respect to the surface. At some time, this would cause the concave-up sections of the field lines to become more shallow and even disappear and therefore cease to be capable of supporting the filament material against gravity (Fan, 2017). Filament material within such a flux rope would then drain from the system as it continued to expand (Mackay *et al.*, 2010). For the event presented in this article, the first obvious observation of material draining is seen to have been initiated on the eastern side at $\approx 01{:}30$ UT. Then, after approximately three hours of sporadic and varied mass motions, the largest mass deposit is observed at $\approx 04{:}30$ UT by SDO/AIA to have propagated towards the eastern footpoint, as shown in Figure 5. Interestingly, this large decrease in mass is shown in Figure 9 to have been initiated at 04:00 UT ($\approx 30$ minutes *prior* to the eruption), although this discrepancy is likely due to difficulties in isolating the initiation of the mass motion amongst all of the additional unrelated intensity variations shown in Figures 5b and c. We therefore conclude that the large mass-unloading began at 04:00 UT. This rapidly reduced the mass contained within the filament, preceding the beginning of the “steep-exponential phase” of the *h–t* profile of Figure 9 and the splitting of the filament into two separate structures.

According to the mass-unloading model described by Klimchuk (2001), the presence of a sufficiently large mass within a non-potential system would allow this system to build free energy without a corresponding expansion, until the removal of the anchoring filament mass. If the mass-unloading were responsible for the change between the two exponential expansions, the ratio between gravitational forces supplied to the flux rope *vs.* the forces acting down on the flux rope from above must indeed be of the order of, or greater than, one, *i.e.* the buoyant flux rope must have been able to overcome the restricting magnetic-tension forces in order for the system to have accelerated. We can explore this through an order-of-magnitude estimation of the magnetic-tension force, as outlined in the derivation presented in Equation 6.2.18 of Aschwanden (2005). The resulting ratio between the gravitational forces and magnetic forces is therefore

7 $$ \frac{ (\Delta \rho ) g}{ \frac{ B^{2}}{ \mu_{0} r_{\mathrm{c}}} }, $$

where $\rho $ is the plasma density, $g$ is the acceleration due to gravity, $\mu_{0}$ is the permeability of free space, and $B$ and $r_{\mathrm{c}}$ are the magnitude of the magnetic field and curvature radius of the overlying loops, respectively, that supply the tension acting down on the flux rope.

The value of mass unloaded was obtained from the STEREO-B/EUVI column number density estimates of Section 4.4. Using the assumption of a filament slab of dimensions $2\times 10^{8}$, $20\times 10^{8}$, and $200\times 10^{8}$ cm, the volume was estimated to be $8\times 10^{27}$ cm^3^, and therefore the change in density was calculated to be $8.25\times 10^{-15}$ g cm^−3^. The magnitude of $B$ for the field overlying the flux rope was estimated by computing a potential-field-source-surface (PFSS) model at 06:04 UT, at the start of the eruption (*cf.* Schrijver and De Rosa, 2003). The PFSS model of the coronal magnetic field was extrapolated using photospheric boundary conditions that were updated in six-hour intervals. Although the extrapolation was carried out on a *post*-eruption photospheric magnetic field, this was deemed to more closely match the photospheric conditions at the time of eruption than those present in the magnetogram taken $\approx \text{five}$ hours *prior* to the eruption. The curvature radius for the overlying field was taken as a range, the lower bound of which was specified as the height at which the filament can be seen at by STEREO-B when the *h–t* profile changes from the shallow-exponential to the steep-exponential phase, therefore $r_{\mathrm{c}} \approx 70\,\text{--}\,90$ Mm, yielding a magnetic-field strength [$B$] for the apex of the potential field overlying the flux rope of between 3 and 2.2 G. This produced a ratio between gravitational and magnetic-tension forces of 1.8 to 4.1, respectively, at 04:40 UT, the change from shallow- to steep-exponential phase. Therefore the height increase associated temporally with the mass-unloading shown in Figure 9 is interpreted as this expansion of the magnetic field due to the weakening of the anchoring force supplied by the large, dense filament. In addition, the gravitational and kinetic energy of the system were calculated to have increased by $1.1\times 10^{28}$ ergs and $4.8\times 10^{26}$ ergs, respectively, due to this unloading of mass.

As previously mentioned, the filament is seen to have split into two distinct structures in the lead-up to its eruption: the dynamic portion that erupts, and the restrained portion that remains. Studies have been carried out to better understand how a filament can split into two during its evolution; Gilbert, Holzer, and Burkepile (2001) described a partial filament eruption and offered an explanation of how the magnetic environment, in their case a flux rope, might evolve and separate during an eruption. In the event that we have presented here, these types of observations are not available for study and so we are not able to test the applicability to their model. However, as both of our split filaments are assumed to involve flux ropes, it is reasonable to assume that this event shares some similarity to theirs. According to Figure 2, the separation can be seen to have occurred by $\approx 05{:}26$ UT, after the initiation of the large mass-unloading. As demonstrated above, it is inferred that the mass-unloading was responsible for the change in the nature of the expansion of the filament from a shallow- to steep-exponential rise. It also appears that the mass-unloading was located at the apex of the dynamic portion of the filament, reducing the anchoring force of this region of the filament to the surface. With no noticeable mass-unloading having occurred within the region that becomes the restrained portion, it is reasonable to assume that this region did not experience the same reduced anchoring force and expansion as the dynamic portion. Therefore, during the expansion of the dynamic portion, it is possible that the magnetic structure underwent some form of vertical reconnection due to the rise, as in the model presented by Gilbert, Holzer, and Burkepile (2001), and this permitted the splitting of the proposed flux rope into two. Interestingly, the material suspended in the restrained portion of the proposed flux rope, in the event presented here, is visibly perturbed during the eruption of the dynamic portion. This perturbed material is seen to then reform the restrained portion of the filament some hours after the eruption, suggesting that the magnetic structure of the restrained portion did not reconfigure to a significant degree during the nearby eruption and perturbation.

Finally, both the monochromatic and polychromatic column number density determination methods were applied to the filament in this investigation. The monochromatic method returns a lower estimate of the column number density (which in itself is already a lower limit) as we have only insufficient data to constrain the filling factor and the foreground emission fraction. Therefore the values calculated are treated as estimates. However, the internal mass structure is well highlighted by examining the results of this method applied to the target at multiple times, and an evolution in mass can be estimated. While the polychromatic method was able to constrain the filling factor and the foreground emission, and hence give a more certain lower limit on the column number density, it is not possible to infer a mass for the whole filament in this instance because of the uncertainty of the unattenuated radiation field behind the filament. The column number density estimates of portions of the filament material derived using the polychromatic method are found to be almost two orders of magnitude greater than for the monochromatic technique. This suggests that either this structure is far more dense than the STEREO-B data indicate, or the filament structure indeed resembled a slab topology, and SDO was simply observing a thicker structure from above than STEREO-B was from the side. A topological description of the slab structure from the magnetic dips in a flux rope may be found in the simulations carried out by authors such as Régnier and Amari (2004), and Hillier and van Ballegooijen (2013). Nevertheless, if the density estimates of the SDO are more representative of the filament, the ratio of gravitational forces to magnetic forces would increase by the corresponding orders of magnitude, indicating that the mass-unloading played a significant role in the final evolution of the filament.

## Conclusions

We have presented a multi-wavelength study of the pre-eruption period of the partial filament eruption on 11 December 2011 using data from two spacecraft. The multiple viewpoints have revealed the height response of the filament due to material dynamics within; a separation otherwise unachievable from a single perspective. Four main stages of evolution were isolated: a stable phase ($12{:}00\,\text{--}\,{\approx}\, 13{:}00$ UT), a rise phase (13:00 – 01:00 UT), a shallow-exponential phase (01:00 – 04:40 UT), and a steep-exponential phase (04:40 onwards). The rise phase temporally coincides with flux cancellation along the PIL below the filament of interest. Similarly to the nearby bipole emergence that was favourably oriented for reconnection with the magnetic arcade overlying and restraining the filament, the proposed flux rope becoming kink-unstable, and the continued expansion of the filament through the solar atmosphere are seen to be contemporaneous, potentially highlighting cancellation, emergence, and kinking as the triggers for the partial eruption (Wang and Sheeley, 1999; Török and Kliem, 2005). The proposed flux rope containing the filament is then seen to have become marginally unstable, demonstrated by a shallow-exponential evolution in the height of the filament at 01:00 UT. Importantly, a large mass deposit beginning at 04:00 UT corresponding to a decrease in filament mass of 70 % (a larger percentage than those reported previously by Bi *et al.*, 2014 and Fan, 2017) yielded a lower-limit ratio range of 1.8 to 4.1 between gravitational forces and magnetic-tension forces. The expansion of the dynamic portion of the filament is then seen to accelerate; we therefore conclude that the observed mass-unloading was responsible for the transition between the two exponential expansions of the filament.

## Electronic Supplementary Material

Below is the link to the electronic supplementary material.

(MP4 22.9 MB)

## Footnotes

1 “Unattenuated radiation field” refers to the emission from behind and in front of the material in question; the radiation field as it would appear to an observer in the absence of the material would not be reduced in intensity by any absorption as there is no material there to absorb, or “attenuate”, the background intensity.
